# Observation of a quantum phase transition in the quantum Rabi model with a single trapped ion

**DOI:** 10.1038/s41467-021-21425-8

**Published:** 2021-02-18

**Authors:** M.-L. Cai, Z.-D. Liu, W.-D. Zhao, Y.-K. Wu, Q.-X. Mei, Y. Jiang, L. He, X. Zhang, Z.-C. Zhou, L.-M. Duan

**Affiliations:** 1 Center for Quantum Information, Institute for Interdisciplinary Information Sciences, Tsinghua University, Beijing, PR China; 2 Department of Physics, Renmin University, Beijing, PR China; 3 Beijing Academy of Quantum Information Sciences, Beijing, PR China

**Keywords:** Physics, Atomic and molecular physics, Optical physics, Quantum physics

## Abstract

Quantum phase transitions (QPTs) are usually associated with many-body systems in the thermodynamic limit when their ground states show abrupt changes at zero temperature with variation of a parameter in the Hamiltonian. Recently it has been realized that a QPT can also occur in a system composed of only a two-level atom and a single-mode bosonic field, described by the quantum Rabi model (QRM). Here we report an experimental demonstration of a QPT in the QRM using a ^171^Yb^+^ ion in a Paul trap. We measure the spin-up state population and the average phonon number of the ion as two order parameters and observe clear evidence of the phase transition via adiabatic tuning of the coupling between the ion and its spatial motion. An experimental probe of the phase transition in a fundamental quantum optics model without imposing the thermodynamic limit opens up a window for controlled study of QPTs and quantum critical phenomena.

## Abstract (web summary)

Quantum phase transition occurs in many-body systems with abrupt changes in the ground state around zero temperature. Here the authors report signatures of quantum phase transition in single trapped ion that can be described using quantum Rabi model.

## Introduction

Quantum phase transitions (QPTs) have become one of the focuses of condensed matter physics. Unlike classical phase transitions that occur at finite temperature, a QPT can occur at zero temperature under quantum fluctuations^1–3^. When a control parameter, such as the external magnetic field or the doping of a component, is scanned across a quantum critical point, the ground state of the system changes abruptly, characterized by a spontaneous symmetry breaking or a change in the topological order^2,4^.

Studies of QPTs usually consider many-body systems in the thermodynamic limit, with the particle number *N* approaching infinity^3^. However, it was recently realized that a QPT can also occur in a small system with only two constituents, a two-level atom and a bosonic mode, described by the quantum Rabi model (QRM)^5–12^ which is one of the simplest models of light-matter interactions. Its Hamiltonian can be expressed as (throughout this paper we set *ℏ* = 1 for simplicity)

1 $${\hat{H}}_{{\rm{QRM}}}=\frac{{\omega }_{{\rm{a}}}}{2}{\hat{\sigma }}_{z}+{\omega }_{{\rm{f}}}{\hat{a}}^{\dagger }\hat{a}+\lambda \left({\hat{\sigma }}_{+}+{\hat{\sigma }}_{-}\right)\left(\hat{a}+{\hat{a}}^{\dagger }\right),$$

where ${\hat{a}}^{\dagger }$ ($\hat{a}$) is the bosonic mode creation (annihilation) operator and ${\hat{\sigma }}_{+}$ (${\hat{\sigma }}_{-}$) is the two-level system raising (lowering) operator; *ω*_a_, *ω*_f_, and *λ* are the atomic transtion frequency, the field mode frequency and the coupling strength between the two subsystems, respectively. This model has been widely studied in multiple paramter regions with many experimental platforms. When ∣*ω*_a_ − *ω*_f_∣ ≪ ∣*ω*_a_ + *ω*_f_∣ and *λ*/*ω*_f_ ≪ 1 are fulfilled, the rotating wave approximation (RWA) can be used to simplify the QRM to the Jaynes–Cummings model (JCM)^13,14^ which has been investigated first in cavity QED^15–17^ and trapped ions^18^, and then in other platforms such as quantum dots^19^ and circuit QED^20,21^. When *λ* becomes comparable to *ω*_a_ + *ω*_f_, the RWA breaks down leading to the ultra-strong coupling regime (*λ*/*ω*_f_ ≳ 0.1) and deep-strong coupling regime (*λ*/*ω*_f_ ≳ 1)^14^. Many exotic dynamical properties in these regimes have been observed recently in a plenty of quantum systems such as circuit QED^22–27^, photonic system^28^, semiconductor system^29,30^, and trapped ions^31^.

In the trapped-ion systems, previous works on the simulation of the QRM have been performed in various regimes. For *ω*_a_ = 0, *ω*_f_ ≠ 0, the QRM reduces to the spin-dependent force Hamiltonian which is crucial in trapped-ion quantum computation^32–35^. For *ω*_a_ ≠ 0, *ω*_f_ = 0, the Dirac equation has been simulated with trapped ions^36,37^. For *ω*_a_ = 0, *ω*_f_ = 0, the coupling-only regime can be realized and it has been exploited to engineer the Schrödinger cat state^38,39^ and the grid state^40,41^. By controlling the experimental parameters, ref. ^31^ has access to the ultra-strong and the deep-strong coupling regimes. However, most of the previous works focus on the evolution dynamics governed by the QRM Hamiltonian in multiple regimes.

Our work realizes the model Hamiltonian in a special parameter region *ω*_a_ ≫ *ω*_f_, which allows the study of a QPT with the phases controlled by the coupling strength *λ* in the QRM. In ref. ^11^, it has been shown that an order parameter, the rescaled photon number in the bosonic mode, is shown to stay zero in the normal phase while acquiring positive values in the superradiant phase with a spontaneous breaking of the *Z*_2_ parity symmetry. The ground state of the system exhibits nonanalytical behavior at the critical point, supporting a second-order phase transition at zero temperature^11^. We experimentally demonstrate this type of QPT without the conventional thermodynamic limit of a large number of particles. Through laser driving near the blue and the red motional sidebands, we use a single trapped ^171^Yb^+^ ion to simulate the QRM Hamiltonian with adjustable parameters^14,31^. We perform a slow quench on the control parameter and measure the average atomic-level population, and the average phonon number as the order parameters on both sides of the transition point. The experiments are repeated for the increasing ratios of *ω*_a_ and *ω*_f_, with the limit *ω*_a_/*ω*_f_ → *∞* analogous to the thermodynamic limit^11^. From the qualitative behavior of the order parameters under the increasing ratios, we obtain strong evidence of the QPT in the QRM, although the ratio parameter is still not large enough for a precise scaling analysis of the critical phenomenon. Our work simulates the QRM in a special parameter region and develops a tool for adiabatic passages that allows the controlled study of a QPT, and showcases the possibility of exploring the universal QPT properties using the trapped-ion system, which has a number of tunable experimental knobs that can be used for a controlled study of the QPT and the critical phenomena under influence of various effects.

## Results

### The quantum critical point in the quantum Rabi model

To study the QPT, the low-energy effective Hamiltonian in the limit *ω*_a_/*ω*_f_ → *∞* has been derived in ref. ^11^. When the control parameter $g\equiv 2\lambda /\sqrt{{\omega }_{{\rm{a}}}{\omega }_{{\rm{f}}}}\ <\ 1$, the effective Hamiltonian in the normal phase is given by ${\hat{H}}_{{\rm{np}}}={\omega }_{{\rm{f}}}{\hat{a}}^{\dagger }\hat{a}-{g}^{2}{\omega }_{{\rm{f}}}{(\hat{a}+{\hat{a}}^{\dagger })}^{2}/4-{\omega }_{{\rm{a}}}/2$ with the qubit frozen in its ground state; and when *g* > 1 we have the effective Hamiltonian in the superadiant phase ${\hat{H}}_{{\rm{sp}}}={\omega }_{{\rm{f}}}{\hat{a}}^{\dagger }\hat{a}-{\omega }_{{\rm{f}}}{(\hat{a}+{\hat{a}}^{\dagger })}^{2}/(4{g}^{4})-{\omega }_{{\rm{a}}}({g}^{2}+{g}^{-2})/4$ in a displaced frame of the bosonic mode, with the qubit ground state now rotated toward the *x*-axis due to its strong coupling to the bosonic mode. This generates non-zero spin and bosonic population in the ground state of the superradiant phase. Hence, we can utilize both the rescaled bosonic mode number (${n}_{{\rm{f}}}\equiv ({\omega }_{{\rm{f}}}/{\omega }_{{\rm{a}}})\langle {\hat{a}}^{\dagger }\hat{a}\rangle$) and the spin population (${n}_{{\rm{a}}}=1+\langle {\hat{\sigma }}_{z}\rangle$) at ground state as the order parameters: in the limit *ω*_a_/*ω*_f_ → *∞*, we have *n*_f_ = 0(*n*_a_ = 0) when *g* < 1 and ${n}_{{\rm{f}}}=({g}^{4}-{g}_{{\rm{c}}}^{4})/(4{g}^{2})({n}_{{\rm{a}}}=1-{g}^{-2})$ for *g* > 1^11,12^.

### Experimental setup

**Fig. 1:**
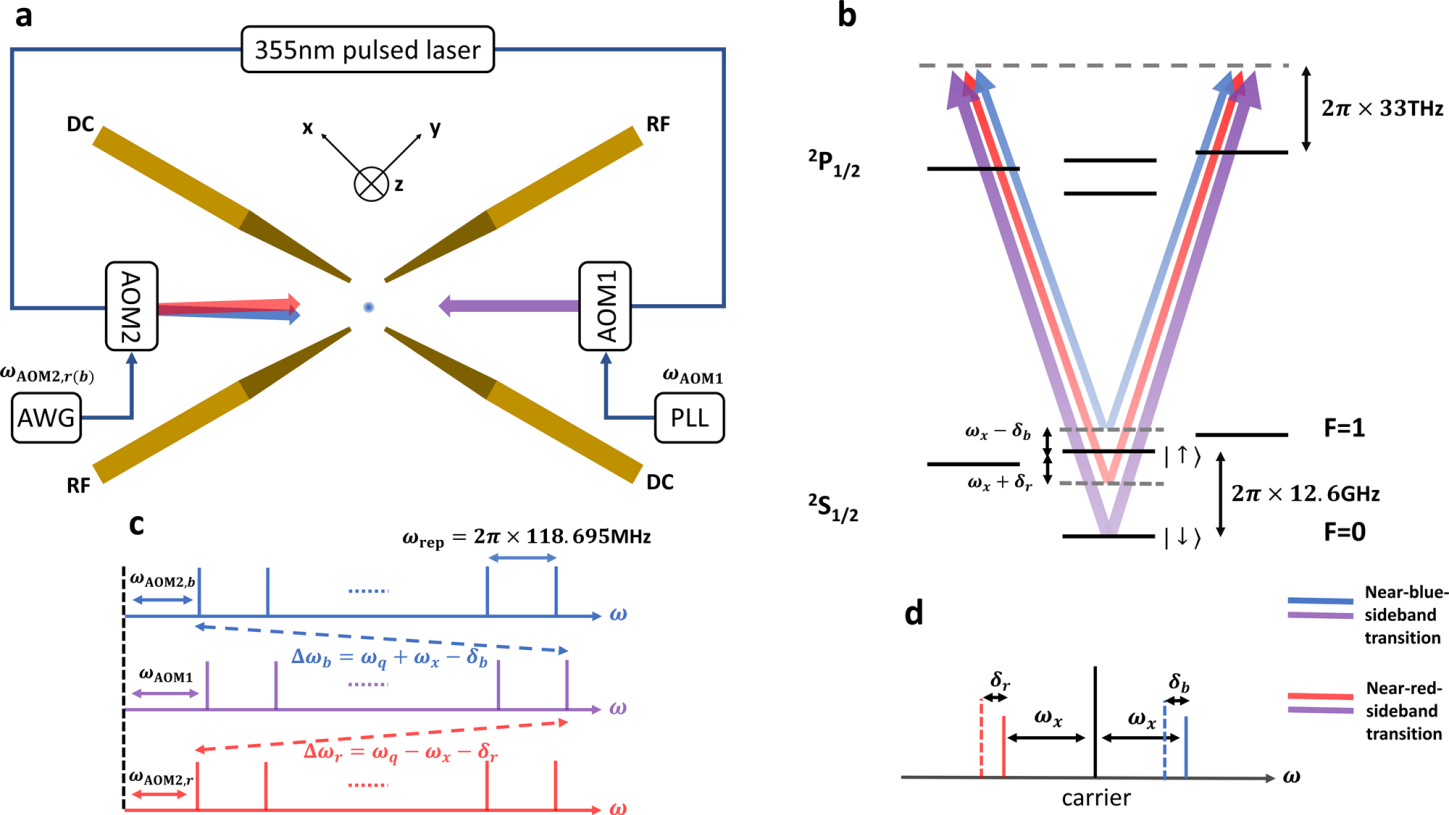
Schematic for experimental observation of QPT in the quantum Rabi model. **a** Schematic experimental setup. The ^171^Yb^+^ ion is confined in the middle of a four-blade Paul trap, with the principal axes of the secular motion along the *x*, *y*, and *z* directions. Two counter-propagating 355 nm pulsed-laser beams are focused on the ion, with a nonzero differential wave vector component along the *x* direction. The two laser beams are controlled by two acousto-optic modulators (AOMs). AOM1 is driven by a radio-frequency (RF) signal from a phase-locked loop (PLL)^56^ and AOM2 is controlled by an arbitrary waveform generator (AWG). **b** Schematic level structure of ^171^Yb^+^. The two qubit states are two ^2^*S*_1/2_ hyperfine ground states $\left|\uparrow \right\rangle =\left|F=1,{m}_{{\rm{F}}}=0\right\rangle$ and $\left|\downarrow \right\rangle =\left|F=0,{m}_{{\rm{F}}}=0\right\rangle$, at the separation *ω*_q_ ≈ 2*π* × 12.6 GHz. The Raman transition is mediated by a virtual level about 2*π* × 33 THz above the ^2^*P*_1/2_ levels. The differential frequencies of the laser beams are tuned close to the blue and the red motional sidebands, i.e., *ω*_x_ − *δ*_b_ and −(*ω*_x_ + *δ*_r_) from the carrier transition. The legend at lower right shows clearly that the purple beam and the blue (red) beam form a near-blue-sideband (near-red-sideband) Raman transition. **c** The 355 nm pulsed laser has a frequency-comb structure^42^ with the repetition rate *ω*_rep_ ≈ 2*π* × 118.695 MHz. With small frequency adjustments in the AOMs, the desired Raman transitions can be achieved between distant teeth of the frequency combs. **d** Relative positions of the carrier transition (black) and two motional sidebands (red and blue) in solid lines and the bichromatic Raman-transition frequencies (red and blue) in dashed lines.

We use a single ^171^Yb^+^ ion confined in a linear Paul trap to simulate the QRM, as shown in Fig. 1a. By performing the Doppler cooling followed by a resolved sideband cooling^18^, the spatial motion of the ion along one of its principal axes *x*, with the frequency *ω*_x_ = 2*π* × 2.35 MHz, is cooled close to the ground state. Its motional degree of freedom can be well described as a quantum harmonic oscillator, and thus serves as the bosonic mode in the QRM. The two hyperfine states in the ground-state manifold ^2^*S*_1/2_ are chosen as the qubit states, i.e., $\left|\uparrow \right\rangle =\left|F=1,{m}_{{\rm{F}}}=0\right\rangle$ and $\left|\downarrow \right\rangle =\left|F=0,{m}_{{\rm{F}}}=0\right\rangle$, with a frequency difference *ω*_q_ ≈ 2*π* × 12.6 GHz as shown in Fig. 1b. We use two counter-propagating 355 nm pulsed-laser beams to manipulate the hyperfine qubit through Raman transition. The pulsed laser has a frequency-comb structure as shown in Fig. 1c, which can help bridge the large frequency gap *ω*_q_ between the two levels^42^; the undesired teeth of the frequency combs can effectively produce a fourth-order AC Stark shift^43^, which we carefully measure and compensate in the experiment (see “Methods” section for more details). Two acousto-optic modulators (AOMs) are used to fine-tune the frequencies and the amplitudes of the laser beams for driving the Raman transition.

The orientation of the laser beams are chosen such that there is a nonzero differential wave vector component Δ*k*_x_ along the *x* axis. Let us first consider a single pair of Raman beams with the frequency and the phase difference Δ*ω* and Δ*ϕ* generating a Rabi frequency Ω. The laser-ion coupling Hamiltonian is given by ${\hat{H}}_{{\rm{couple}}}={{\Omega }}\cos ({{\Delta }}{k}_{{\rm{x}}}\cdot \hat{x}-{{\Delta }}\omega \cdot t+{{\Delta }}\phi ){\hat{\sigma }}_{{\rm{x}}}$^44^, where $\hat{x}={x}_{0}(\hat{a}+{\hat{a}}^{\dagger })$ is the ion-position operator with *x*_0_ being the ground state wave-packet width. Considering the Lamb-Dicke approximation $\eta \sqrt{2\bar{n}+1}\ll 1$ where *η* ≡ Δ*k*_x_*x*_0_ is the Lamb–Dicke parameter and $\bar{n}$ is the average phonon number of the motional state (see Supplementary Information for more details about the correction of the Lamb–Dicke approximation), we transfer ${\hat{H}}_{{\rm{couple}}}$ into the interaction picture of the uncoupled Hamiltonian ${\hat{H}}_{0}={\omega }_{q}{\hat{\sigma }}_{{\rm{z}}}/2\,+{\omega }_{{\rm{x}}}{\hat{a}}^{\dagger }\hat{a}$, and get the interaction Hamiltonian ${\hat{H}}_{{\rm{r}}}=(\eta {{{\Omega }}}_{{\rm{r}}}/2)(\hat{a}{\hat{\sigma }}_{+}{e}^{{\rm{i}}{\delta }_{{\rm{r}}}{\rm{t}}}+{\hat{a}}^{\dagger }{\hat{\sigma }}_{-}{e}^{-{\rm{i}}{\delta }_{{\rm{r}}}{\rm{t}}})$ if the frequency difference Δ*ω* is tuned close to the red motional sideband with *δ*_r_ = *ω*_q_ − *ω*_x_ − Δ*ω*, and ${\hat{H}}_{{\rm{b}}}=(\eta {{{\Omega }}}_{{\rm{b}}}/2)({\hat{a}}^{\dagger }{\hat{\sigma }}_{+}{e}^{{\rm{i}}{\delta }_{{\rm{b}}}{\rm{t}}}+\hat{a}{\hat{\sigma }}_{-}{e}^{-{\rm{i}}{\delta }_{{\rm{b}}}{\rm{t}}})$ when Δ*ω* is tuned close to the blue sideband with *δ*_b_ = *ω*_q_ + *ω*_x_ − Δ*ω*.

In order to construct the QRM Hamiltonian, we employ the bichromatic Raman beams as shown in Fig. 1 driving the red and the blue sidebands simultaneously^32–34^ using the specific implementation proposed and realized recently in ref. ^14,31^, as shown in Fig. 1b. If we set the two Rabi frequencies to be the same Ω_r_ = Ω_b_ = Ω (in the experiment we can calibrate them such that the imbalance ∣Ω_r_ − Ω_b_∣/∣Ω_r_ + Ω_b_∣ ≤ 2%), the resulting Hamiltonian is ${\hat{H}}_{{\rm{rb}}}\,=(\eta {{\Omega }}/2){\hat{\sigma }}_{+}(\hat{a}{e}^{{\rm{i}}{\delta }_{{\rm{r}}}t}+{\hat{a}}^{\dagger }{e}^{{\rm{i}}{\delta }_{{\rm{b}}}{\rm{t}}})+h.c.$, which corresponds to the interaction picture Hamiltonian with respect to the uncoupled Hamiltonian $\hat{H}^{\prime} =-({\delta }_{{\rm{b}}}+{\delta }_{{\rm{r}}}){\hat{\sigma }}_{{\rm{z}}}/4-({\delta }_{{\rm{b}}}-{\delta }_{{\rm{r}}}){\hat{a}}^{\dagger }\hat{a}/2$^14^,

2 $$\begin{array}{ll}{\hat{H}}_{{\rm{rb}}}^{{\rm{I}}}=\frac{{\delta }_{{\rm{b}}}+{\delta }_{{\rm{r}}}}{4}{\hat{\sigma }}_{{\rm{z}}}+\frac{{\delta }_{{\rm{b}}}-{\delta }_{{\rm{r}}}}{2}{\hat{a}}^{\dagger }\hat{a}\\ \qquad\qquad\quad\ \, +\, \frac{\eta {{\Omega }}}{2}\left({\hat{\sigma }}_{+}+{\hat{\sigma }}_{\!-}\right)\left(\hat{a}+{\hat{a}}^{\dagger }\right).\end{array}$$

We clearly see the transformed Hamiltonian is exactly the QRM Hamiltonian, if we identify *ω*_a_ = (*δ*_b_ + *δ*_r_)/2, *ω*_f_ = (*δ*_b_ − *δ*_r_)/2 and *λ* = *η*Ω/2. From our definition, the control parameter is $g\equiv 2\lambda /\sqrt{{\omega }_{{\rm{a}}}{\omega }_{{\rm{f}}}}=2\eta {{\Omega }}/\sqrt{{\delta }_{{\rm{b}}}^{2}-{\delta }_{{\rm{r}}}^{2}}$. Since the uncoupled Hamiltonian $\hat{H}^{\prime}$ commutes with our desired observables, the spin (${\hat{\sigma }}_{{\rm{z}}}$) and the phonon (${\hat{a}}^{\dagger }\hat{a}$) population, their measurements will not be affected by this transformation^14^. By controlling the experimental parameters *δ*_b_, *δ*_r_, and Ω, we can achieve the simulation in the regime *ω*_a_ ≫ *ω*_f_ where an observation of a QPT is possible.

### Observation of quantum phase transition from the spin population

To observe the QPT from the normal phase to the superradiant phase in the QRM Hamiltonian, we consider two measurable order parameters, the spin-up state population $(1+\langle {\hat{\sigma }}_{{\rm{z}}}\rangle )/2$^12^ and the average phonon number $\langle {\hat{a}}^{\dagger }\hat{a}\rangle$^11^. As the control parameter *g* rises from zero to above the quantum critical point, the *Z*_2_ parity symmetry is broken, and these two values at the ground state will accordingly increase from zero to a non-zero value. However, it is hard to prepare the ground state of a general Hamiltonian^45^, and since the energy gap closes at the quantum critical point, we are not able to adiabatically scan the control parameter across this point without generating the quasi-particle excitations into the system^11^. Therefore, in this experiment we perform slow quench on the control parameter as suggested by ref. ^11^, and compare the measured values with the theoretical predictions.

First, we set *δ*_b_ = 2*π* × 52.0 kHz and *δ*_r_ = 2*π* × 48.0 kHz, which corresponds to a ratio *R* ≡ *ω*_a_/*ω*_f_ = 25 between the atomic transition frequency and the field mode frequency in the QRM. Under this finite ratio, the energy gap at the quantum critical point becomes finite which is around 0.4*ω*_f_ = 2*π* × 0.8 kHz^11^, indicating that the quench time should at least be 1.25 ms such that the prepared state does not deviate too much from the true ground state. After sideband cooling, we initialize the ion in the ground state $\left|\downarrow ,n=0\right\rangle$. Then we linearly increase the sideband Rabi frequency such that ${{{\Omega }}}_{{\rm{SB}}}(t)\equiv \eta {{\Omega }}(t)={{{\Omega }}}_{\max }t/{\tau }_{{\rm{q}}}$ where ${{{\Omega }}}_{\max }=2\pi \times 14.2\ {\rm{kHz}}$ and the quench time *τ*_q_ = 2 ms are two pre-determined parameters. In other words, the time to reach the critical point ${{{\Omega }}}_{{\rm{SB}}}^{{\rm{c}}}=\sqrt{{\delta }_{{\rm{b}}}^{2}-{\delta }_{{\rm{r}}}^{2}}/2=2\pi \times 10\ {\rm{kHz}}$ is about 1.4 ms. We expect the quantum state of the system to follow the slow quench of the control parameter $g(t)={{{\Omega }}}_{{\rm{SB}}}(t)/{{{\Omega }}}_{{\rm{SB}}}^{{\rm{c}}}$. Hence with a duration time *t*, we generate the target state under a specific coupling strength of the QRM and measure the order parameters.

**Fig. 2:**
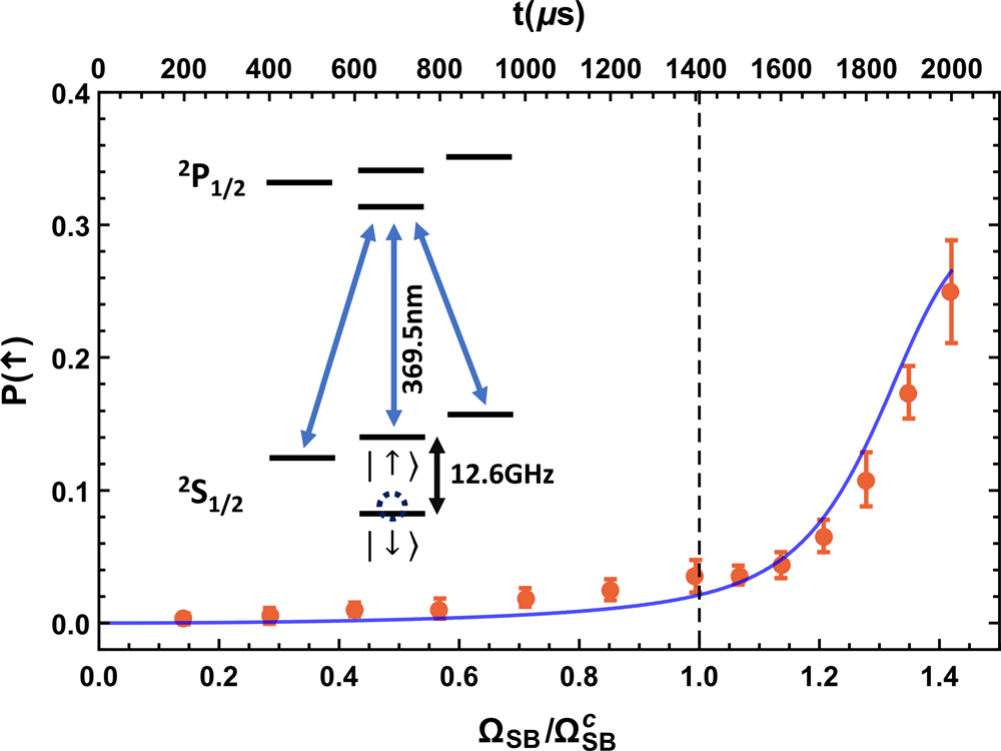
Spin-up state population versus sideband Rabi frequency. By setting *δ*_b_ = 2*π* × 52.0 kHz and *δ*_r_ = 2*π* × 48.0 kHz, we keep the ratio parameter *R* = *ω*_a_/*ω*_f_ = 25 fixed. As we increase the sideband Rabi frequency Ω_SB_ (bottom axis) linearly with time (top axis), i.e., ${{{\Omega }}}_{{\rm{SB}}}\,=\,{{{\Omega }}}_{\max }t/{\tau }_{{\rm{q}}}$ where ${{{\Omega }}}_{\max }=2\pi \times 14.2\ {\rm{kHz}}$ and the quench time *τ*_q_ = 2 ms are two pre-determined parameters, the control parameter $g(t)\,=\,2{{{\Omega }}}_{{\rm{SB}}}(t)/\sqrt{{\delta }_{{\rm{b}}}^{2}-{\delta }_{{\rm{r}}}^{2}}$ goes up accordingly. With a duration time *t*, we prepare a target state under *g*(*t*) and measure the spin-up state population by florescence detection. Every orange dot is the average of 20 rounds of measurements of the spin-up state population, corrected by subtracting the 1.0% dark-state detection error as the background; the error bar is estimated as one standard deviation of the 20-round outcomes (see Supplementary Information for more details about the error bar estimation). For each round of measurement, we repeat the experiment sequence for 500 shots and take the average. The blue curve is the theoretical value by directly solving the time-dependent Schrödinger equation under the QRM Hamiltonian. The vertical dashed line is an indication of the quantum critical point *g*_c_ = 1 (corresponding to ${{{\Omega }}}_{{\rm{SB}}}^{{\rm{c}}}=2\pi \times 10\ {\rm{kHz}}$). The inset shows the florescence detection scheme of ^171^Yb^+^ ions^46^.

The spin-up state population can be measured by a resonant driving on the $|{\,}^{2}{S}_{1/2},F=1\rangle \to |{\,}^{2}{P}_{1/2},F=0\rangle$ cyclic transition of the ^171^Yb^+^ ion and a detection of the scattered photon counts^46^. The result is shown in Fig. 2. Every orange data point is the average of 20 rounds of measurements of the spin-up state population and has been corrected by subtracting the 1.0% dark-state detection error, which arises from the small residual off-resonant coupling of the detection laser to the bright state^46^ as the background. For each round of measurement, the outcome is acquired by averaging over 500 shots of the experiment sequence. The error bar is estimated by one standard deviation of the 20 rounds. We clearly observe the increase of the order parameter $(1+\langle {\hat{\sigma }}_{{\rm{z}}}\rangle )/2$ after the quantum critical point (the vertical dashed line in Fig. 2) despite the relatively low sharpness due to the finite ratio parameter *R*, which agrees well with the numerical simulation (the blue curve in Fig. 2 from numerically solving the time-dependent Schrödinger equation of the QRM Hamiltonian).

### Observation of quantum phase transition from the phonon number

Next we consider another order parameter, the average phonon number. After the slow quench of the QRM Hamiltonian, a short optical pumping pulse of 5 μs is applied to pump the internal state of the ion (qubit state) into $\left|\downarrow \right\rangle$^46^ with negligible effect on the motional state (phonon state) population. Then, we drive the blue-sideband transition between $\left|\downarrow ,n\right\rangle$ and $\left|\uparrow ,n+1\right\rangle$ (*n* = 0, 1, …) for various time interval *t*. By fitting the resultant spin-up state population, we can reconstruct the population of different phonon states, thus calculating the average phonon number^17,18,38,39,47–51^.

**Fig. 3:**
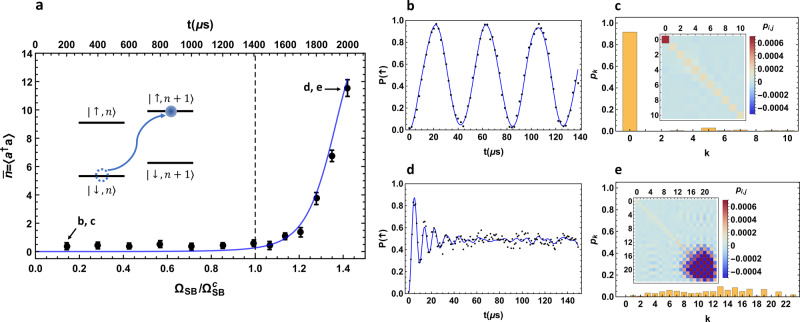
Average phonon number versus sideband Rabi frequency. Again we set *δ*_b_ = 2*π* × 52.0 kHz and *δ*_r_ = 2*π* × 48.0 kHz, thus the ratio parameter *R* = *ω*_a_/*ω*_f_ = 25. With the same quench process as above, we prepare the target states and measure the corresponding average phonon numbers. **a** Each black dot is a measured average phonon number for a specific ground state. Its value and the error bar are determined according to **b**–**e**. The blue curve is the theoretical result by solving the time-dependent Schrödinger equation. The inset shows the blue sideband scheme for analyzing the phonon number distribution: before the measurement, we optically pump the spin state into $\left|\downarrow \right\rangle$^46^ with tiny influence to the phonon state population; then we drive the blue sideband transition for various time interval and fit the obtained spin-up state population to extract the phonon distribution. For the leftmost data point in the normal phase, **b** presents the experimental data (black dots, averaged over 200 shots) and the fitted curve (blue line), and **c** shows the fitted population *p*_k_ (*k* = 0, 1, …) with the covariance matrix shown in the inset. The error bar in **a** is computed from this covariance matrix as one standard deviation for the average phonon number. Similarly **d** and **e** show the results for the rightmost data point in **a** in the superradiant phase. More details can be found in the “Methods” section.

With the same experimental parameters as above, the results are shown in Fig. 3. Each black dot in Fig. 3a is the calculated average phonon number from the phonon population distribution with the error bar estimated by one standard deviation. In Fig. 3b we show an example for the blue sideband signal of the leftmost data point in Fig. 3a. The measured spin-up state population is fitted by the blue curve to give the phonon state population {*p*_k_} (*k* = 0, 1, …) with a suitable truncation. The fitting result is shown in Fig. 3c with a covariance matrix (inset) representing the correlation between different *p*_k_’s, from which we further deduce the standard deviation of the average phonon number, assuming a joint Gaussian distribution^52^. More details can be found in the “Methods” section. As we can see, for this data point we get a very low average phonon number, consistent with the fact that it is deep in the normal phase. Similarly, Fig. 3d, e show the results for the rightmost data point in Fig. 3a. Here, we get much faster oscillation at the beginning of the blue sideband data owing to the much higher phonon number population (the sideband Rabi oscillation frequency $\sim \sqrt{n+1}\eta {{\Omega }}$) in the superradiant phase, as well as much faster decay since the phonon number has a wider distribution. In this case we get larger uncertainty in each fitted *p*_k_. However, they are strongly correlated as shown by the off-diagonal elements of the covariance matrix (inset of Fig. 3e), and we still get a reasonable error bar for the average phonon number. Finally, in Fig. 3a we further compare the measured average phonon number with the theoretical values from numerically solving the time-dependent Schrödinger equation. Again these results agree well within the error bars.

It should be pointed out that the fourth order AC Stark shift induced by the laser beams is not zero in our setup^43^, and will increase as we gradually turn up the coupling strength of the QRM in the above experiments. Therefore, they cannot be compensated by a static frequency shift in the laser beams, but require a dynamic compensation by phase modulation of the laser as shown in the “Methods” section. Also note that for our slow quench dynamics to maintain quantum coherence, the total quench time *τ*_q_ should be shorter than the motional decoherence time *τ*_d_ of the trapped ion. The motional coherence of our system is largely affected by the 50 Hz noise from the AC power line. Therefore we use a line-trigger to lock the experimental sequence to the AC signal from the power line, which extends the motional decoherence time to over 5 ms.

### Scaling of the order parameter with respect to various experimental parameters

**Fig. 4:**
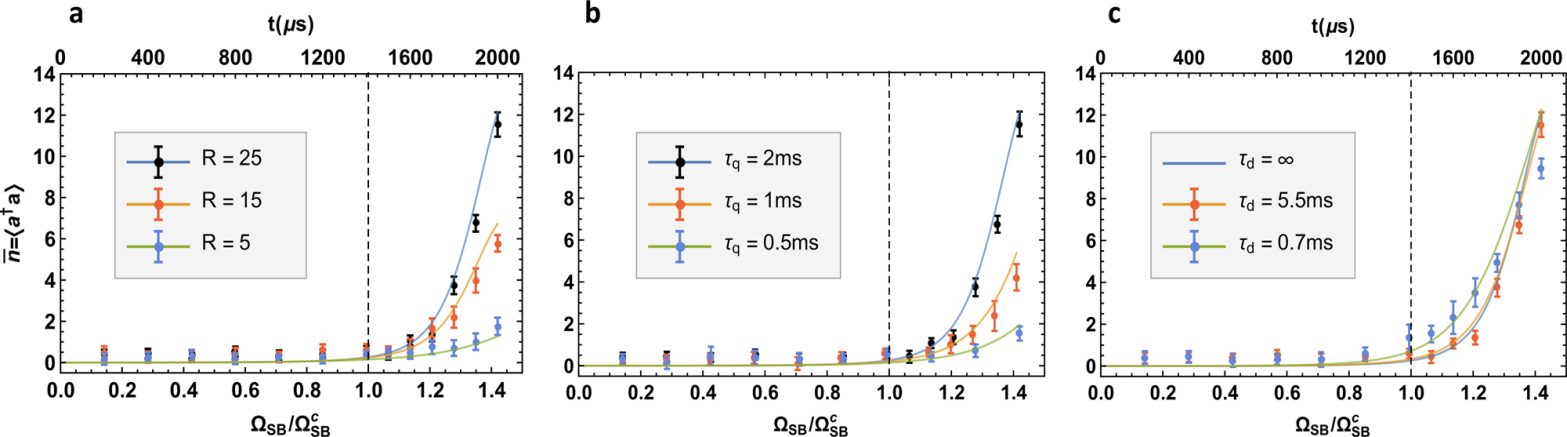
Average phonon number versus sideband Rabi frequency under different experimental parameters. Each dot is an average phonon number measured in the same way as in Fig. 3, with the error bar representing one standard deviation. In **a**–**c**, we vary the ratio parameter *R*, the total quench time *τ*_q_ and the motional decoherence time *τ*_d_, respectively, while keeping the other parameters the same as those in Fig. 3. **a**. We keep ${{{\Omega }}}_{{\rm{SB}}}^{{\rm{c}}}=2\pi \times 10\ {\rm{kHz}}$ and *τ*_q_ = 2 ms. Then we need *δ*_b_ = 2*π* × 41.3 kHz and *δ*_r_ = 2*π* × 36.1 kHz for *R* = 15 and *δ*_b_ = 2*π* × 26.8 kHz and *δ*_r_ = 2*π* × 17.9 kHz for *R* = 5. **b** We keep ${{{\Omega }}}_{{\rm{SB}}}^{{\rm{c}}}=2\pi \times 10\ {\rm{kHz}}$, *R* = 25, but use different quench time *τ*_q_. **c** We keep ${{{\Omega }}}_{{\rm{SB}}}^{{\rm{c}}}=2\pi \times 10\ {\rm{kHz}}$, *R* = 25 and *τ*_q_ = 2 ms, but vary the motional decoherence time *τ*_d_ by turning on (*τ*_d_ = 5.5 ms) or off (*τ*_d_ = 0.7 ms) the locking of the experimental sequence to the 50 Hz reference. The curves in **a** and **b** are from numerical simulation without considering the motional decoherence, similar to the *τ*_d_ = *∞* curve in **c**. The other two curves in **c** include the motional decoherence effect by numerically solving a Lindblad master equation (see “Methods” section for more details). The difference between *τ*_d_ = *∞* and *τ*_d_ = 5.5 ms is very small for the quench time *τ*_q_ = 2 ms, thus justifies our simplification of *τ*_d_ = *∞* for **a**, **b** and the previous numerical simulations.

Finally we consider the scaling of the order parameter with respect to different experimental parameters. For this purpose, the average phonon number is the preferred observable because it can vary in a wider range than the spin-up state population. Our results are summarized in Fig. 4 where we change the ratio parameter *R*, the total quench time *τ*_q_ and the motional decoherence time of the ion *τ*_d_, while keeping the other parameters the same. Figure 4a considers different ratios *R* = (*δ*_b_ + *δ*_r_)/(*δ*_b_ − *δ*_r_) by keeping the critical sideband Rabi frequency ${{{\Omega }}}_{{\rm{SB}}}^{{\rm{c}}}=2\pi \times 10\ {\rm{kHz}}$ fixed. Hence we can deduce ${\delta }_{b(r)}={{{\Omega }}}_{{\rm{SB}}}^{{\rm{c}}}(\sqrt{R}\pm 1/\sqrt{R})$ from the ratio parameter *R*. As expected, the sharpness of the curve and the final average phonon number are positively correlated with the ratio parameter, and approach nonanalytical behavior in the limit *R* → *∞* (see Supplementary Information for a further discussion about the finite-ratio scaling). In Fig. 4b, we vary the quench time *τ*_q_ to study its effect on the order parameter. A shorter quench time leads to a larger deviation from the adiabatic evolution, thus the prepared state has larger deviation from the true ground state. Only for long enough quench time can the prepared states have large enough overlap with the real ground states, hence show the clear evidences of the QPT. In Fig. 4c we study the influence of finite motional decoherence time *τ*_d_ of the trapped ion. To keep the quantum nature of the system during the slow quench dynamics, the quench time should be within the coherence time of the system. As is mentioned above, the motional coherence of our trap is largely affected by the 50 Hz noise from the AC power line. By locking the experimental sequence to the 50 Hz reference, the coherence time is above 5 ms; while if we turn off the locking, the coherence time will drop below 1 ms. This phenomenon is also reported in the ref. ^53^. We conduct the experiments with the locking turned on and off, respectively. As expected, the sharpness of the curve reduces for shorter coherence time. The results agree well with the theoretical prediction for a motional decoherence time *τ*_d_ = 5.5 ms and *τ*_d_ = 0.7 ms, respectively from solving the Lindblad master equation (see “Methods” section). We also perform a simulation using the Schrödinger equation without considering any decoherence, which is labeled as *τ*_d_ = *∞*. This curve is very close to that for *τ*_d_ = 5.5 ms, which justifies our numerical simulation using Schrödinger equation in Figs. 2, 3a, 4a, b, for *τ*_*q*_ ≤ 2 ms. We also notice that the heating of the motional mode and the decoherence of the qubit state are potential sources of errors in the experiments. However, in our system the heating rate measured by the standard method^54,55^ is well below 50 quanta/s, and the qubit coherence time measured by the Ramsey method using Raman transition is greater than 50 ms, which is mainly limited by the coherence of the PLL^56^. Both have negligible effect on the measured order parameters as discussed in “Methods” section.

## Discussion

To sum up, we have successfully observed a QPT from the normal phase to the phonon superradiance phase associated with the QRM simulated by a single trapped ion. Through slow quench dynamics, we measure the spin-up state population and the average phonon number as the order parameters and observe them changing from near zero to large values, when the control parameter is tuned across the quantum critical point. For the average phonon number, the change becomes sharper when the ratio parameter increases, analogous to approaching closer to a thermodynamic limit. The strong controllability of the trapped-ion system also allows us to vary the experimental parameters and study their influence on the phase transition. We also note that in the ref. ^12^, a method to observe the universal scaling with spin-up state population was proposed. However, considering some technical difficulties, it is not possible for our system to observe the critical phenomena currently (see Supplementary Information for more discussions about this). To further study the finite-ratio scaling, we will either need to reduce the experimental noise and to upgrade the experimental setup to get more accurate results near the critical point for larger frequency ratio *R*; or we may need to develop different scaling methods which use data points farther away from the critical point. Our work is a first step towards the more detailed studies of the QPT in the QRM, including the critical dynamics and the universal scaling^11,12^. With reservoir engineering^51,57^, it is also possible to observe the dissipative phase transition in the QRM^58^. Besides, our method can be directly extended to study the QPT in the many-body version of the QRM, i.e., the Dicke model^5,59,60^ when we increase the number of the trapped ions.

## Methods

### AC Stark shift compensation

Our 355 nm pulsed laser has a frequency comb structure with a repetition rate *ω*_rep_ ≈ 2*π* × 118.695 MHz and a bandwidth of about 200 GHz. It can be used to bridge the transition between the two qubit levels with a frequency difference around *ω*_q_ ≈ 2*π* × 12.6 GHz, without the need of large frequency shifts between the two Raman beams^42^. In Fig. 1a, suppose AOM1 introduces a frequency shift of *ω*_AOM1_, which is dynamically varied to compensate the fluctuation of the repetition rate *ω*_rep_^56^, and AOM2 leads to a frequency shift *ω*_AOM2,*r*(*b*)_ for the red (blue) component of the bichromatic laser beams. The closest differential frequencies to the sideband transitions will be Δ*ω*_r(b)_ = *n* × *ω*_rep_ + *ω*_AOM1_ − *ω*_AOM2,r(b)_ with *n* = 107, the span number of the frequency-comb pairs as shown in Fig. 1c.

As we have mentioned in the main text, when tunning the sideband Rabi frequency from zero to a specific value, the AC Stark shift induced by the off-resonant coupling of the undesired frequency-comb pairs will also increase continuously. This is a common shift to *δ*_r_ and *δ*_b_, which changes *δ*_r_ + *δ*_b_ and hence the ratio parameter *R*. For the 355 nm pulsed laser we use, when the sideband Rabi frequency is set to 2*π* × 14.2 kHz, the AC Stark shift can reach over 2*π* × 10 kHz measured by the standard Ramsey method^61^. Such a large shift has non-negligible effect on the order parameters and must be compensated during the slow quench dynamics. Before each round of experiment, we calibrate the AC Stark shift Δ_ac_ under the QRM Hamiltonian with different sideband Rabi frequencies Ω_SB_ and fit it according to ${{{\Delta }}}_{{\rm{ac}}}=\alpha {{{\Omega }}}_{{\rm{SB}}}^{2}$ where *α* is a proportionality constant. Then when performing the slow quench experiment, we correct the frequency of the blue (red) component in the bichromatic beams as *ω*_b(r)_(*t*) = *ω*_b(r)_(0) + Δ_ac_(*t*), to make the detuning *δ*_b(r)_ fixed. This can be realized by phase modulating the driving RF signals on AOM2, which can be conveniently implemented by an AWG as shown in Fig. 1a with a pre-determined waveform loading to its memory. The waveform for the pulse is given by $A(t)\cos ({\omega }_{{\rm{AOM2}},r(b)}t-\mathop{\int}\nolimits_{0}^{t}{{{\Delta }}}_{{\rm{ac}}}(t){\rm{d}}t)$, where *ω*_AOM2,r(b)_ is a pre-set driving frequency of AOM2 at the beginning of the experiment and the driving amplitude *A*(*t*) ∝ Ω_SB_(*t*) is also calibrated before the experiment.

### Phonon number distribution measurement

To measure the phonon number of a quantum state of the spin-phonon system, we trace out the spin part by optically pumping it to $\left|\downarrow \right\rangle$^46^ within a duration of 5 μs so that its influence to the motional state can be neglected. Then we apply a blue sideband pulse with various duration *t* and measure the resultant spin-up state population *P*_*↑*_(*t*). It can be fitted by^18,31,50^

3 $${P}_{\uparrow }(t)=\frac{1}{2}\left[1-\mathop{\sum }\limits_{k = 0}^{{k}_{\max }}{p}_{{\rm{k}}}{e}^{-{\gamma }_{k}t}\cos ({{{\Omega }}}_{{\rm{k,k}}+1}t)\right],$$

where *p*_k_ is the occupation of the phonon number state $\left|k\right\rangle$, *γ*_k_ is a number-state-dependent empirical decay rate of the Rabi oscillation, where we adopt a commonly used form *γ*_k_ ∝ (*k* + 1)^0.7^^18,31,50^, ${{{\Omega }}}_{{\rm{k,k}}+1}=\sqrt{k+1}{{{\Omega }}}_{{\rm{SB}}}$ is the number-state-dependent sideband Rabi frequency, and ${k}_{\max }$ is the cutoff in the phonon number. If the hyperparameter ${k}_{\max }$ in the fitting model is too small, we will lose the high-phonon population and thus limited to a small average phonon number; however, if ${k}_{\max }$ is chosen too large, the uncertainty in the fitting will increase because we need to fit more parameters, and the risk of misjudgement of high-phonon population from the noise of the blue-sideband signals will also increase (see Supplementary Information for more details about the choice of ${k}_{\max }$).

After fitting the phonon state population $P={({p}_{0},{p}_{1},\cdots )}^{{\rm{T}}}$ with its covariance matrix Σ, we can compute the average phonon number $\bar{n}=N\cdot P$ where *N* = (0, 1, …) is a row vector representing the phonon number basis. Assuming the fitted parameters follow a joint Gaussian distribution^52^ (see Supplementary Information for more details about this assumption), we can estimate the variance of $\bar{n}$ as ${\sigma }_{\bar{n}}^{2}=N{{\Sigma }}{N}^{{\rm{T}}}$.

### Error analysis and numerical simulation

To consider the motional decoherence effect, we numerically solve the master equation with the Lindblad superoperator $L[\hat{O}]\hat{\rho }\,\equiv\, \hat{O}\hat{\rho }{\hat{O}}^{\dagger }\,-\,{\hat{O}}^{\dagger }\hat{O}\hat{\rho }/2\,-\,\hat{\rho }{\hat{O}}^{\dagger }\hat{O}/2$ of dephasing type^62^: $\dot{\hat{\rho }}(t)\,=\,-i[\hat{H},\hat{\rho }(t)]\,+L[\sqrt{2{{{\Gamma }}}_{{\rm{m}}}}{\hat{a}}^{\dagger }\hat{a}]\hat{\rho }$, where Γ_m_ = 1/*τ*_d_ is the dephasing rate with the decoherence time *τ*_d_. In Fig. 4c with the line-trigger on (off), we set *τ*_d_ = 5.5 ms (0.7 ms), which is within the range of our daily measurement (see Supplementary Information for more detials about the motional coherence measurement), to fit the experimental data.

For the motional heating and the qubit decoherence, we add the Lindblad superoperators $L[\sqrt{\gamma {n}_{{\rm{th}}}}{\hat{a}}^{\dagger }]+L[\sqrt{\gamma ({n}_{{\rm{th}}}+1)}\hat{a}]$^62^ and $L[\sqrt{2{{{\Gamma }}}_{{\rm{q}}}}{\hat{\sigma }}_{+}{\hat{\sigma }}_{-}]$^63^, respectively, where *γn*_th_ ≈ *γ*(*n*_th_ + 1) is the motional heating rate which is below 50 s^−1^ and Γ_q_ is the qubit decoherence rate which is below 20 s^−1^ in our system. As we have mentioned in the main text, the effects of these two terms are negligible from numerical simulation. All the Lindblad superoperators we used in the master equation just represent the results in the lab frame (describing the experimental decay), and does not represent decay in the simulated system frame (describing the QRM decay).

The fluctuation of the trap frequency (motional mode frequency *ω*_x_), which is within 2*π* × 150 Hz after applying the RF power stabilization^64^, can be the main error source on the ratio parameter *R*, because the trap frequency fluctuation is asymmetrical for *δ*_r_ and *δ*_b_ (see Fig. 1d), causing *δ*_b_ − *δ*_r_ to change, thus the ratio parameter. Under 2*π* × 150 Hz trap frequency fluctuation, the uncertainty for *R* = 25, 15, 5 are ±1.7, ±0.82, and ±0.16, respectively. Other sources of errors can be from the phonon number fitting beacuse some noise in the blue-sideband signals may be incorrectly recognized as a high-phonon population and cause the fitting error, and from the fluctuation of the AC Stark shift due to the fluctuation of the laser repetition rate and the laser intensity. Consider a 1% sideband Rabi freqeuncy fluctuation (i.e., 1% of 2*π* × 14.2 kHz for maximal estimation) and 2*π* × 30 Hz fluctuation of the repetition rate, the standard deviation of the fluctuated AC Stark shift from a theoretical calculation^43^ can reach about 2*π* × 400 Hz. Under this value, the ratio parameter uncertainty for *R* = 25, 15, 5 are ±0.20, ±0.15, and ±0.09, respectively.

## Supplementary information

Supplementary Information

## Data Availability

The data that support the findings of this study are available from the corresponding authors upon reasonable request.

## Code availability

The code used for numerical simulations is available from the corresponding authors upon reasonable request.
