# Supplementary material for: Observation of a quantum phase transition in the quantum Rabi model with a single trapped ion
Source: Nat Commun. 2021 Feb 18;12:1126. doi: 10.1038/s41467-021-21425-8 (PMC7893029; doi:10.1038/s41467-021-21425-8)
Supplement: Supplementary file 1 — Supplementary Information [file 41467_2021_21425_MOESM1_ESM.pdf]

Supplementary information for
Observation of a quantum phase transition in the quantum Rabi model with a single trapped ion

M.-L. Cai*[1], Z.-D. Liu*[1], W.-D. Zhao*[1], Y.-K. Wu[1], Q.-X. Mei[1],
Y. Jiang[1], L. He[1], X. Zhang[2,1], Z.-C. Zhou[1,3], L.-M. Duan†[1]
[1] *Center for Quantum Information, Institute for Interdisciplinary Information Sciences, Tsinghua University, Beijing 100084, PR China*
[2] *Department of Physics, Renmin University, Beijing 100084, PR China and*
[3] *Beijing Academy of Quantum Information Sciences, Beijing 100193, PR China*

NOTE ON THE ERROR BAR ESTIMATION

Error bar estimation in the spin population experiment

In the spin population experiment, there are mainly two types of experimental noise we are considering: one is the intrinsic quantum fluctuation and the other is the extrinsic fluctuation of control parameters and environmental parameters. During one round of the experiment, the system is relatively stable and we are mainly concerned with the quantum projection noise [1]. It arises because the quantum state is not an eigenstate of the observable, say, the spin-up state population and thus by repeating the experiment we get different outcomes even if we prepare the same quantum state. This noise can be suppressed by increasing the number of measurements. By averaging over 500 shots in each experimental round, we get the average spin-up state population with the quantum projection noise suppressed to $1/\sqrt{500}$, which is small compared with other experimental noise.

On the other hand, the prepared quantum states can differ due to the long-term fluctuation of control parameters and environmental parameters. This noise cannot be suppressed by increasing the number of measurements and we regard this as the dominant error source in our experiment. These effects include fluctuation in laser intensity, laser repetition rate, temperature, air pressure, etc. Therefore, we conduct the experiment for 20 rounds, each at a different time with the time interval on the order of several minutes. We then use the standard deviation of the 20-round outcomes to estimate the error bar.

Error bar estimation in the phonon number experiment

When estimating the error bar of the average phonon number, we need to make an assumption about the distribution of the experimental noise. Under the common assumption of independent and identically distributed Gaussian noise of the experimental data, it can be shown that the fitted parameters also follow a joint Gaussian distribution (see e.g. Theorem 2.1 of Ref. [2].), which is what we use in this work. We want to emphasize that this assumption is used in lots of experiments when extracting parameters by fitting the experimental data, and is implicitly used in many scientific computing softwares like MATLAB when fitting parameters.

NOTE ON THE CHOICE OF THE $k_{\max}$ IN THE PHONON NUMBER DISTRIBUTION FITTING

We use the lowest cutoff number that can ensure the total occupation of all the Fock states to be above 95% as $k_{\max}$ in the phonon number distribution fitting. We take the phonon number distribution of the state with the largest average phonon number in this experiment as an example to show how we choose a proper $k_{\max}$. In Fig. S1a, the extracted average phonon number is 11.54 ± 0.71 while the total occupation $\sum_{k=0}^{k_{\max}} p_k$ is around 95.6% with a cutoff number 23 (which can be seen from the horizontal axis). When we continue to increase the cutoff number to 24 (Fig. S1b) and 25 (Fig. S1c), the results of the phonon number distribution are nearly the same with the total

* These authors contribute equally to this work
† Corresponding author: lmduan@tsinghua.edu.cn

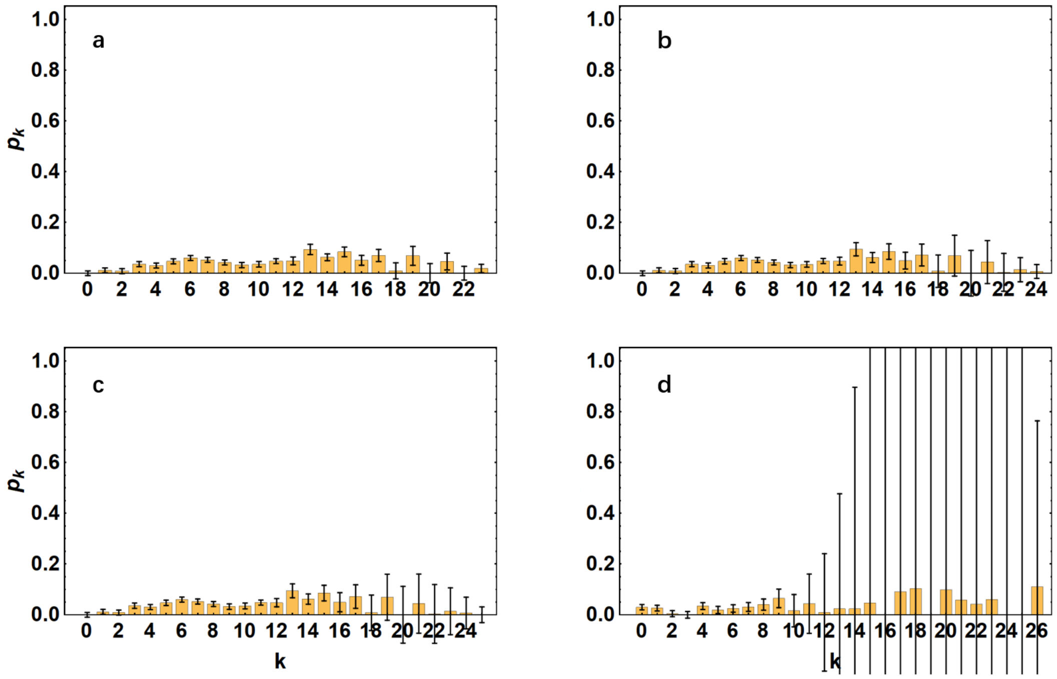

FIG. S1: **Phonon number distribution with different cutoff number $k_{\max}$.** The phonon number distribution with cutoff number $k_{\max} = 23$ in **a**, $k_{\max} = 24$ in **b**, $k_{\max} = 24$ in **c** and $k_{\max} = 24$ in **d**. The error bar is one standard deviation from the fitting program. The extracted average phonon number is 11.54 ± 0.71 while the total occupation $\sum_{k=0}^{k_{\max}} p_k$ is around 95.6% in **a**. The results of the phonon number distribution are nearly the same with the total occupation around 95.9% and the average phonon number $11.63 \pm 0.73\,(0.74)$ in **b** and **c**. However, when the cutoff number is set to 26 (**d**), the phonon number distribution dramatically changes and the error bar of the occupation of the Fock states after $|12\rangle$ becomes very large, indicating that overfitting occurs.

occupation around 95.9% and the average phonon number $11.63 \pm 0.73\,(0.74)$. However, when the cutoff number is set to 26 (Fig. S1d), the phonon number distribution dramatically changes and the error bar of the occupation of the Fock states after $|12\rangle$ becomes very large, indicating that overfitting occurs. Also, according to the numerical simulation, the total occupation number above the Fock state $|24\rangle$ (including $|24\rangle$) is only 0.13%, contributing an average phonon number around 0.03 to this state, which is much smaller than the error due to the fitting of about 0.7. Hence, this also justifies the choice of the cutoff number 23. The reason why the total occupation in our fitting is only around 95% may be due to the state preparation and measurement error (SPAM error) during the blue-sideband pulse analysis (described in Methods). For instance, even if we can ideally prepare the phonon ground state, i.e. only the Fock state $|0\rangle$ is occupied and the only non-zero occupation is p_0. We can easily see that p_0 is the contrast of the sinusoidal spin-up state population curve used to extract the occupation number. However, due to the SPAM error, the contrast must be less than 1. In our system, the SPAM error is around 2% (an average of 1% dark-state detection error and 3% bright-state detection error), which means the contrast of the spin-up state population curve is only 96%. This explains the relatively low total occupation.

NOTE ON THE CORRECTION FOR THE LAMB-DICKE APPROXIMATION

All of our discussions in the main text are based on the condition that the single trapped ion is in the Lamb-Dicke regime. In this regime, the extension of the ion's wave function is much smaller than the laser's wavelength, or this limitation can be written as $\eta\sqrt{2\bar{n}+1} \ll 1$ [3], where η is the Lamb-Dicke parameter and $\bar{n}$ is the average phonon number of the motional state. In our system, the Lamb-Dicke parameter is around 0.07. However, in our experiment, the maximum average phonon number exceeds ten, which means $\eta\sqrt{2\bar{n}+1}$ is around 0.3, making the non-linear terms of η a non-negligible effect to the entire model Hamiltonian. In the following, we consider the corrections to the numerical results of the two order parameters due to the non-linear effect.

When we consider the non-linear terms, the total Hamiltonian of the QRM simulated by a single trapped ion reads

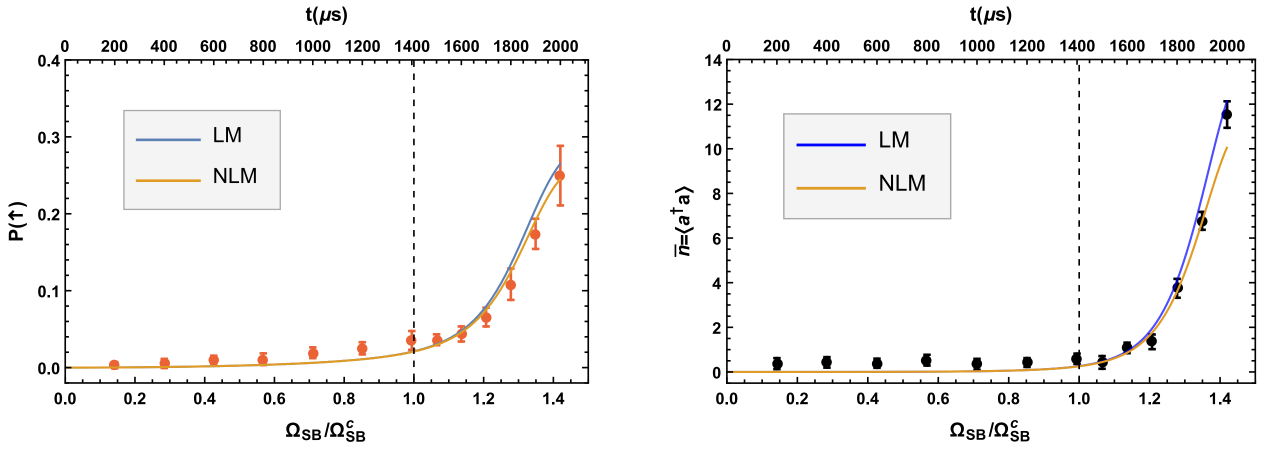

FIG. S2: **The spin-up state population and the average phonon number versus the sideband Rabi frequency with/without non-linear effect.** Here we set the experimental parameters the same as the main text with $\delta_b = 2\pi \times 52.0\,\mathrm{kHz}$, $\delta_r = 2\pi \times 48.0\,\mathrm{kHz}$, thus the ratio parameter $R = 25$. The total quench time $\tau_q = 2\,\mathrm{ms}$ with the sideband Rabi frequency increases linearly from zero to $\Omega_{\max} = 2\pi \times 14.2\,\mathrm{kHz}$. The Lamb-Dicke parameter is $\eta = 0.07$. **a** and **b** are Fig. 2 and Fig. 3a in the main text with an additional numerical result of the non-linear QRM, respectively. We can see clearly that in the normal phase, the phonon number is small enough that both the two order parameters in the non-linear model (NLM) show good consistency with those in the linear model (LM). In the superradiant phase, with the increase of the average phonon number, the non-linear effect becomes more and more significant and causes a non-negligible deviation of the two order parameters between the NLM and the LM.

[4]:

$$\hat{H}_{\mathrm{NQRM}} = \frac{\omega_{\mathrm{a}}}{2}\hat{\sigma}_z + \omega_{\mathrm{f}}\hat{a}^\dagger\hat{a} + \lambda\left(\hat{\sigma}_+ + \hat{\sigma}_-\right)\left(\hat{f}\hat{a} + \hat{a}^\dagger\hat{f}\right), \tag{S1}$$

where the non-linear effect is embodied in the function [5]

$$\hat{f}(\hat{a}, \hat{a}^\dagger) = e^{-\eta^2/2}\sum_{l=0}^{\infty}\frac{\left(-\eta^2\right)^l}{l!(l+1)!}\hat{a}^{\dagger l}\hat{a}^l. \tag{S2}$$

When we only consider the first expansion term, i.e. $l = 0$ and neglect the term $e^{-\eta^2/2}$, the Hamiltonian reduces to the linear QRM. Here, we implement a numerical simulation additionally considering an $l = 1$ term.

As shown in Fig. S2, with the same experimental parameters as in the main text, we simulate the effect on the spin-up state population and the average phonon number during the quench dynamics. As we can see, in the normal phase, the phonon number is small enough that both the two order parameters in the non-linear model (NLM) show good consistency with those in the linear model (LM). In the superradiant phase, with the increase of the average phonon number, the non-linear effect becomes more and more significant. In our simulation, we find that the maximum relative deviation of the average phonon number between the NLM and the LM ($|\bar{n}_{\mathrm{LM}} - \bar{n}_{\mathrm{NLM}}|/\bar{n}_{\mathrm{LM}}$) is about 17 %. However, the deviation near the critical point is only about 2 %, which is small enough compared with other errors discussed in the Methods.

In conclusion, the non-linear terms in the simulated QRM causes a small but non-negligible deviation when the average phonon number is large ($\gtrsim 10$). However, because they are still smaller than the leading term, we expect the qualitative behavior of the quantum phase transition, in particular the universal class near the phase transition point, to be unaffected.

NOTE ON THE SCALING ANALYSIS

Scaling analysis with spin population

We note in Ref. [6], spin population is used to analyze the scaling effect of the QPT in the QRM. However, some of the experimental parameters and conditions in Ref. [6] are rather stringent for our system. There are mainly three

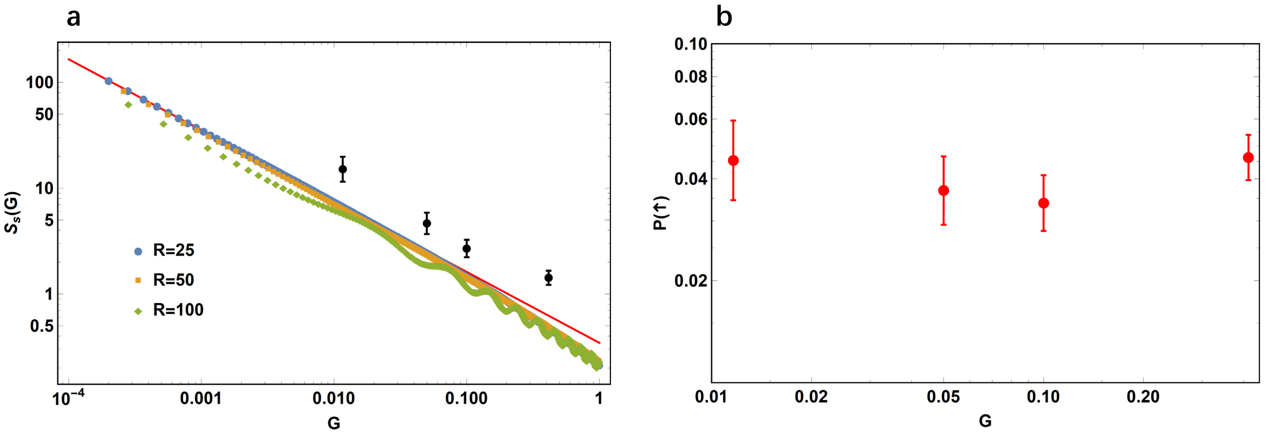

FIG. S3: **Scaling analysis with spin-up state population. a**. The $S_s(G)$-G plot, where $S_s(G) \equiv 2P(\uparrow)|g-1|^{-1}$ and $G \equiv R|g-1|^{3/2}$ with g the coupling strength and $P(\uparrow)$ the spin-up state population. The blue, yellow and green points are numerical simulation results and the red curve is an analytical line with a slope $-2/3$, which is a critical exponent [6]. The numerical results agree well with the analytical line except the numerical result with $R = 100$. This is because when the ratio R is too large, the carrier term in the trapped-ion simulation will cause the simulated Hamiltonian to deviate from the real QRM model [6]. The black points with error bar are calculated from the experimental results with $R = 25$. The error bar is estimated as the error bar of the spin-up state population $P(\uparrow)$ multiplied by the corresponding $|g-1|^{-1}$. **b**. The four experimental data presented in **a** near the critical point $g_c = 1$, with their raw values of $(g, P(\uparrow))$ being $(0.994, 0.0453 \pm 0.0123)$, $(0.984, 0.0369 \pm 0.0085)$, $(0.975, 0.0339 \pm 0.0064)$, $(1.065, 0.0462 \pm 0.0071)$ respectively. Although in the log-plot, these points seem to nicely follow a trend with the red line and their error bars are not that large compared to the difference of these points, the difference between the raw data points is on the same order as the raw data error bars. We believe the trend indicated by the four black points is just dominated by the dependence of $S_s(G)$ and G on $|g-1|$.

conditions that are currently not achievable in our system. First, in Ref. [6] the bosonic mode frequency $\tilde{\omega}_0/2\pi$ ($\omega_f/2\pi$ in our notation) is set to 200 Hz to realize large frequency ratio R of 50 to 400 under realistic coupling strength. This is comparable to the trap frequency fluctuation (around 150 Hz) and even smaller than the fluctuation of the estimated AC Stark shift (around 400 Hz, see Methods) in our system, and therefore will lead to large error. Second, under such large frequency ratios, the required adiabatic evolution time of about 250 ms is too long compared to our qubit coherence time under Raman laser of about 40 to 60 ms. Finally, Ref. [6] proposes a standing wave configuration for the laser beams in order to suppress the influence of the carrier term under large frequency ratio, but our setup uses a traveling wave configuration which is more common in current ion trap experiments. The standing wave configuration needs four laser beams instead of the two beams in the traveling wave configuration. It is not easy to change our current configuration to four beams. We believe these technical challenges can be overcome with (1) choosing a more appropriate bosonic mode frequency (e.g 1 kHz) with a still achievable coupling strength (300 kHz carrier Rabi rate); (2) suppressing the system noises by improving the RF amplitude stabilization system and choosing a more appropriate repetition rate of the Raman laser; (3) improving the coherence time of the system (including the motional coherence time).

Given the current condition of our system, we choose a moderate ratio $R = 25$ to implement the spin population experiment and show the overall behavior in Fig. 2 in the main text. Here we further supplement some experimental data around the critical point together with a numerical simulation according to Ref. [6]. We summarize the results in Fig. S3a. The figure is a $S_s(G)$-G plot where $S_s(G) \equiv 2P(\uparrow)|g-1|^{-1}$ and $G \equiv R|g-1|^{3/2}$ with g the coupling strength and $P(\uparrow)$ the spin-up state population. The blue, yellow and green points are numerical simulation results and the red curve is an analytical line with a slope $-2/3$ (note that according to Ref. [6], the asymptotic behavior of $S_s(G)$ is $\lim_{G\to 0} S_s(G) \propto G^{-2/3}$, i.e. there is a universal critical exponent -2/3). The numerical results agree well with the analytical line except the numerical result with $R = 100$. This is because when the ratio R is too large, the carrier term in the trapped-ion simulation will cause the simulated Hamiltonian to deviate from the real QRM model and this is why Ref. [6] propose a standing-wave laser configuration to suppress the influence of the carrier term. The black points with error bar are calculated from the experimental results with $R = 25$. The error bar is estimated as the error bar of the spin-up state population $P(\uparrow)$ (which is the raw data taken from the experiment) multiplied by the corresponding $|g-1|^{-1}$ which is supposed to be accurate. The four experimental points (from left to right) are all very close to the critical point $g_c = 1$, where their raw data values of $(g, P(\uparrow))$ are $(0.994, 0.0453 \pm 0.0123)$,

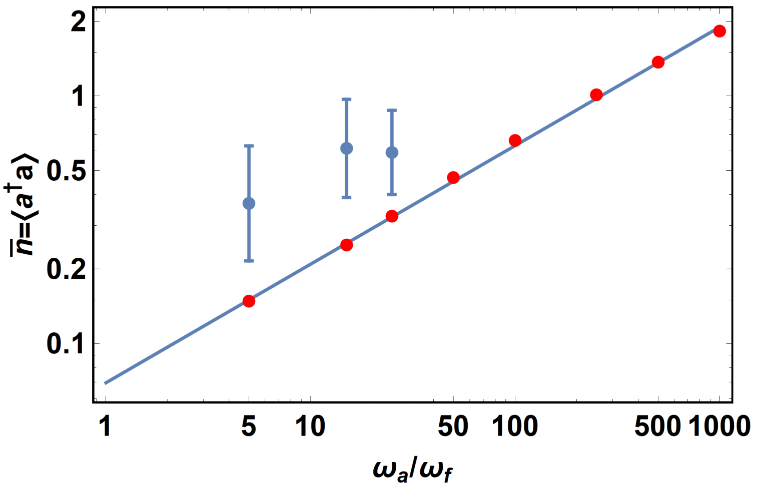

FIG. S4: **Finite-ratio scaling of the average phonon number near the critical point.** The red points are the numerical results with the system size indicated by the ratio $R = \omega_{\mathrm{a}}/\omega_{\mathrm{f}}$ ranging from 5 to 1000 and the fitting result shows that the slope of the fitting linear line is 0.48. The blue points with error bar are the experiment results. Under the current achievable ratio R, the difference between these points is on the same order of magnitude as the error bar, indicating they are vulnerable to the experimental noises.

$(0.984, 0.0369 \pm 0.0085)$, $(0.975, 0.0339 \pm 0.0064)$, $(1.065, 0.0462 \pm 0.0071)$ respectively. Although in the log-plot, these points seem to nicely follow a trend with the red line and their error bars are not that large compared to the difference of these points, the difference between the raw data points is on the same order as the raw data error bars (see Fig. S3b). We believe the difference between the raw data points can be easily washed out due to experimental noises (e.g. the fluctuations of trap frequency and AC Stark shift) because they are too close to the same point. Hence, we believe the trend indicated by the four black points is just dominated by the dependence of $S_s(G)$ and G on $|g-1|$. In conclusion, the precision of the current experiment prevents us from observing the universal scaling law with spin population.

Scaling analysis with average phonon number

We present a numerical simulation of the finite-ratio scaling of the average phonon number near the critical point $g_{\mathrm{c}} = 1$ and show the result in Fig. S4. The red points are the numerical results with the system size (indicated by the ratio $R \equiv \omega_{\mathrm{a}}/\omega_{\mathrm{f}}$) ranging from 5 to 1000 and the fitting result shows that the slope of the fitting line is 0.48. The blue points with error bar are the experimental results. Under the current achievable ratio R, the difference between these points is on the same order of magnitude as the error bar, indicating they are vulnerable to the experimental noises. Thus these points cannot be used to extract the critical exponent. Also, we note that the fitted slope of 0.48 from the numerical simulation data actually deviates from the true critical exponent 1/3 in the regime $R \to \infty$ in analytic calculation (see Ref. [7]). In order to see this precise exponent, the ratio R in the numerical simulation needs to exceed 10^5. Due to such large ratio, the adiabatic ground state preparation may need a duration orders of magnitude larger than the coherence time of the system. Hence it is not achievable for our system currently to observe the precise scaling effect and to extract the critical exponent with average phonon number. We can only observe the overall behavior of the phonon number variation curves with three different ratios (5, 15 and 25), and as expected the curve becomes sharper with larger ratio (see Fig. 4a in the main text).

NOTE ON THE RAMSEY INTERFEROMETRIC MEASUREMENT FOR MOTIONAL COHERENCE

We use the commonly used Ramsey method [8] to measure the motional coherence time with or without the line-trigger on. We apply two $pi/2$ blue-sideband pulses with a time interval τ in between and then measure the spin population. By varying the time interval τ, we obtain the Ramsey fringes shown in Fig. S5. We fit the result by an

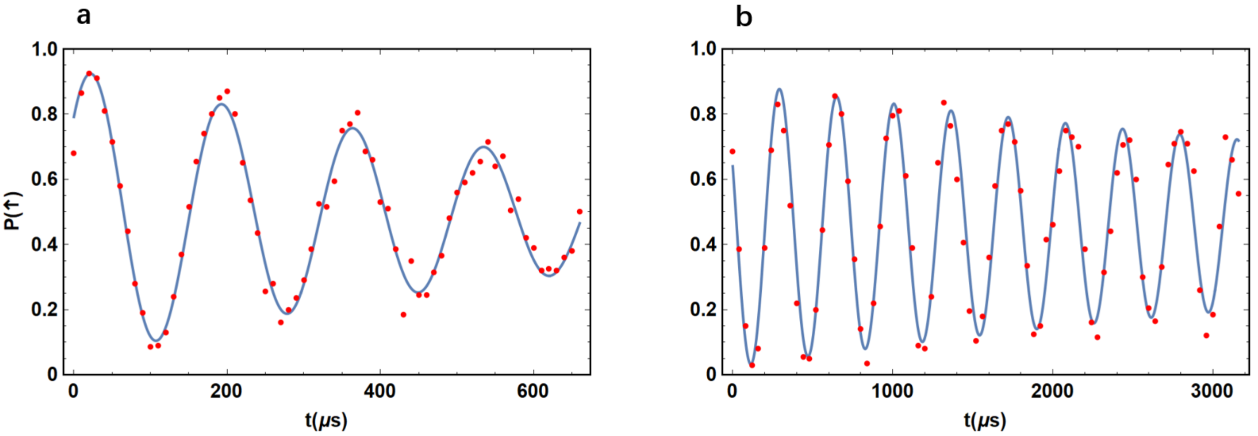

FIG. S5: **The motional coherence time measured by the Ramsey method.** **a**. Without the line-trigger on, the Ramsey fringes decay fast and the estimated coherence time is around 0.7 ms. **b**. With the line-trigger on, the Ramsey fringes decay much slower and the estimated coherence time is around 5.5 ms

attenuated sinusoid curve $Ae^{-t/\tau_d}\cos(\omega t+\phi)$ where A, τ_d, ω and ϕ are the fitting parameters. The coherence time τ_d is extracted from the fitted curve. In Fig. S5a, the estimated coherence time is around 0.7 ms and in b, the estimated coherence time is around 5.5 ms. As we can see, the line-trigger can significantly improve the motional coherence time.

[1] W. M. Itano, J. C. Bergquist, J. J. Bollinger, J. M. Gilligan, D. J. Heinzen, F. L. Moore, M. G. Raizen, and D. J. Wineland, "Quantum projection noise: Population fluctuations in two-level systems," Phys. Rev. A **47**, 3554–3570 (1993).

[2] G. A. F. Seber and C. J. Wild, *Nonlinear regression* (Hoboken, NJ: Wiley-Interscience, 2003).

[3] D. Leibfried, R. Blatt, C. Monroe, and D. Wineland, "Quantum dynamics of single trapped ions," Rev. Mod. Phys. **75**, 281–324 (2003).

[4] Xiao-Hang Cheng, Iñigo Arrazola, Julen S. Pedernales, Lucas Lamata, Xi Chen, and Enrique Solano, "Nonlinear quantum rabi model in trapped ions," Phys. Rev. A **97**, 023624 (2018).

[5] W. Vogel and R. L. de Matos Filho, "Nonlinear jaynes-cummings dynamics of a trapped ion," Phys. Rev. A **52**, 4214–4217 (1995).

[6] Ricardo Puebla, Myung-Joong Hwang, Jorge Casanova, and Martin B. Plenio, "Probing the dynamics of a superradiant quantum phase transition with a single trapped ion," Phys. Rev. Lett. **118**, 073001 (2017).

[7] Myung-Joong Hwang, Ricardo Puebla, and Martin B. Plenio, "Quantum phase transition and universal dynamics in the rabi model," Phys. Rev. Lett. **115**, 180404 (2015).

[8] Norman F. Ramsey, "A molecular beam resonance method with separated oscillating fields," Phys. Rev. **78**, 695–699 (1950).
